# DCM2Net: an improved face recognition model for panoramic stereoscopic videos

**DOI:** 10.3389/frai.2024.1295554

**Published:** 2024-06-11

**Authors:** Dalei Zhang, Wee Hoe Tan, Yuanyuan Wei, Chung Keat Tan

**Affiliations:** ^1^Faculty of Social Sciences and Liberal Arts, UCSI University, Kuala Lumpur, Malaysia; ^2^School of Geography and Tourism, Zhengzhou Normal University, Zhengzhou, China; ^3^Faculty of Medicine and Health Sciences, UCSI University, Kuala Lumpur, Malaysia

**Keywords:** face recognition, DCM2Net, panoramic stereo video, feature extraction, PSD dataset

## Abstract

The panoramic stereo video has brought a new visual experience for the audience with its immersion and stereo effect. In panoramic stereo video, the face is an important element. However, the face image in panoramic stereo video has varying degrees of deformation. This brings new challenges to face recognition. Therefore, this paper proposes a face recognition model DCM2Net (Deformable Convolution MobileFaceNet) for panoramic stereo video. The model mainly integrates the feature information between channels during feature fusion, redistributes the information between channels in the deeper part of the network, and fully uses the information between different channels for feature extraction. This paper also built a panoramic stereo video live system, using the DCM2Net model to recognize the face in panoramic stereo video, and the recognition results are displayed in the video. After experiments on different datasets, the results show that our model has better results on popular datasets and panoramic datasets.

## 1 Introduction

Face recognition technology is a biometric recognition technology based on face features, which has broad development prospects. In recent years, with the development of deep learning and neural networks, face recognition technology has made a lot of research results and become one of the important research directions in the field of computer vision. The face recognition algorithm based on the convolutional neural network can accurately learn the features of face recognition after a large number of data training, so as to achieve better results in face verification.

**Figure 1:**
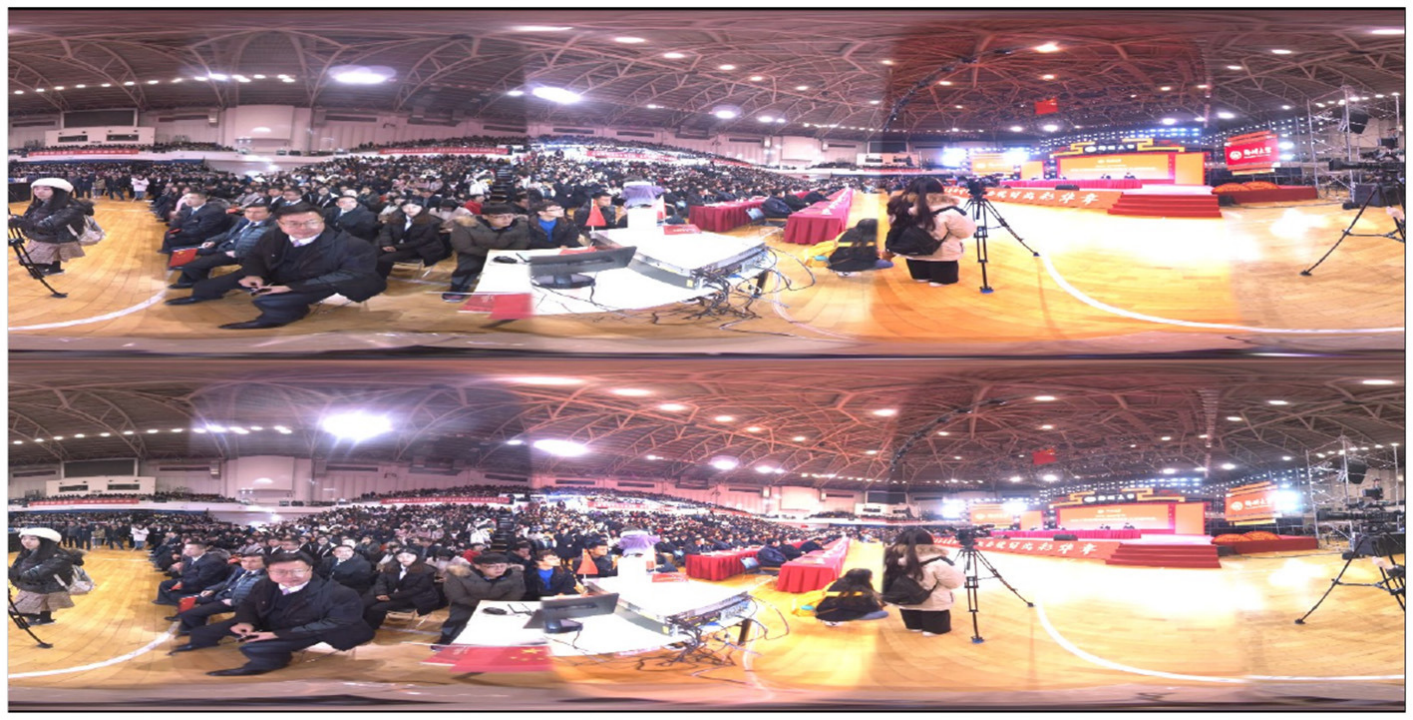
Screenshot of the party scene based on panoramic stereoscopic video live streaming.

Panoramic stereo video is an immersive audio-visual experience. Combining panoramic photography and stereo imaging technology presents a realistic and immersive scene for the audience. This video format can let the audience feel the feeling of being in it as if they were in a real environment. It has important application value in live sports broadcasts, video entertainment, and other fields, as shown in Figure 1. With the increase in panoramic video, face recognition based on panoramic video has become a research hotspot.

Due to the special structure of the fisheye lens, there will be barrel distortion in the picture. Therefore, when the face appears in this part of the region, it will also produce varying degrees of deformation. Binocular stereo video simulates the effect observed by human eyes by placing two cameras in parallel at a short distance apart and shooting videos at different angles at the same time. In panoramic stereo video, the same person's face will be captured by different cameras from different angles, such as different angles, different occlusion ranges, etc. To solve these problems, this paper proposes a new face recognition model DCM2Net. The network uses deformable convolution to extract features of deformed faces. In traditional convolutional neural networks, the convolutional kernel can only sample the input feature map at fixed locations, resulting in performance improvements that require training large-scale datasets and increasing model parameters. In contrast, deformable convolution is more flexible and can adaptively sample the input feature map so that the sampled region is no longer restricted to a regular rectangular sampling grid. This enables the model to better capture local features and variations in the image, thus solving the face deformation problem more effectively. In this model, the features of left and right eye face images are fused, and the information between channels is redistributed in the deeper part of the network. By making full use of the information between different channels for feature extraction, DCM2Net achieves higher accuracy and faster recognition efficiency in panoramic stereo video. In addition, we constructed a panoramic stereo video live system and constructed a new panoramic stereo dataset (PSD) based on panoramic video to test the performance of the model. The main contributions of this work are as follows:

- To address the issue of image distortion caused by barrel distortion in panoramic video, this paper proposes a new face recognition model called DCM2Net that leverages the left and right eye face image features.
- This model uses deformable convolution to extract deformable face features and improve the accuracy of face recognition in panoramic videos.
- We design a panoramic stereo video live broadcast system and apply the DCM2Net model proposed in this paper to perform face recognition within the system.

## 2 Related work

In recent years, deep convolutional neural networks have been increasingly used in face recognition. Using neural networks to extract facial features has achieved better results than traditional methods.

Taigman et al. (2014) achieved a good recognition effect by constructing a neural network with 120 million parameters and using large-scale data for training. Zhou et al. (2015) proposed a ten-layer simple convolutional neural network to further increase the accuracy. The DeepId series (Sun et al., 2014a,b, 2015a,b) improves the classic neural network structure of image classification and applies it to face recognition to improve performance. The traditional softmax loss is widely used in image classification. But the softmax loss function itself is used to solve the problem of multi-classification and is not optimized for the hidden feature layer. Often the directly trained features do not have good generalization ability, and the effect is not good in face recognition. In response to the shortcomings of softmax loss, many researchers have improved it (Liu et al., 2016; Wen et al., 2016; Liu W. et al., 2017; Wang et al., 2018). Among them, Deng et al. (2019) proposed to maximize the classification boundary in the angular space and achieved excellent recognition accuracy. Schroff et al. (2015) improved the loss function and applied the triplet loss function to the network. Recently, lightweight networks have become the focus of research. Iandola et al. (2016) reduce the network construction by reducing the parameters of the convolution kernel. Fran (2017) network uses Depthwise Separable Convolutions to cut down parameters.

There is more and more research on face recognition in panoramic video (Liu Y.-F. et al., 2017; Zhang et al., 2021; Kocacinar et al., 2022; Shahin et al., 2022; Hakobyan, 2023; Liu et al., 2023; Perroni Filho et al., 2023a,b). Fu et al. (2019) create a fisheye face image dataset by sampling patches from face images applying fisheye-looking distortion to them, and using it to train the model. However, the panoramic image needs to be processed multiple times on the fisheye images that compose a single panoramic image, which will cost a lot of money. Therefore, it is more convenient to process the equirectangular projection. Yang et al. (2018) proposes that extracting features on the equirectangular projection of panoramic images, but the task is object detection rather than face recognition. Therefore, the existing network structure needs to be improved, and a new network structure is proposed for face recognition of fisheye lens panoramic video.

## 3 Method and experiments

This section will introduce the DCM2Net model framework and the construction of a panoramic stereoscopic video live-streaming system.

### 3.1 Deformable convolution

We use deformable convolution to solve the face deformation problem by firstly computing the offset of each point on the input feature map by conventional convolution, then applying the offset to the input feature map, and finally extracting features from the offset feature map. That is, the deformable convolution deforms the sampled points of the input feature map instead of deforming the convolution kernel. Suppose matrix *X* (Equation 1) is the sampled region of the input feature map and matrix *W* (Equation 2) is the weight points of the 3 × 3 convolution kernel:


(1) $$\begin{array}{l} X=\left\{\begin{array}{ccc} x_{\left(-1,-1\right)} & x_{\left(0,-1\right)} & x_{\left(1,-1\right)}\\ x_{\left(-1,\;0\right)} & x_{\left(0,\;0\right)} & x_{\left(1,\;0\right)}\\ x_{\left(-1,\;1\right)} & x_{\left(0,\;1\right)} & x_{\left(1,\;1\right)} \end{array}\right\} \end{array}$$

(2) $$\begin{array}{l} W=\left\{\begin{array}{ccc} w_{\left(-1,-1\right)} & w_{\left(0,-1\right)} & w_{\left(1,-1\right)}\\ w_{\left(-1,\;0\right)} & w_{\left(0,\;0\right)} & w_{\left(1,\;0\right)}\\ w_{\left(-1,\;1\right)} & w_{\left(0,\;1\right)} & w_{\left(1,\;1\right)} \end{array}\right\} \end{array}$$

For the ordinary convolution operation, the output *y* (Equation 3) is


(3) $$\begin{array}{l} y_{\left(0,0\right)}=\underset{i,j}{\sum }w_{\left(i,j\right)}\times x_{\left(i,j\right)} \end{array}$$

where i, j traverse the set (-1,0,1).

For deformable convolution, the offset (Δ*x*_(*i, j*)_, Δ*y*_(*i, j*)_) of each point of the input feature map is first calculated by ordinary convolution, where Δ*x*_(*i, j*)_ and Δ*y*_(*i, j*)_ denote the offset distance of the horizontal and vertical coordinates at the current sampling point *x*_(*i, j*)_, respectively, and then the offset is applied to the input feature map as the fresh input feature map, and the output *y* (Equation 4) is


(4) $$\begin{array}{l} y_{\left(0,0\right)}=\underset{i,j}{\sum }w_{\left(i,j\right)}\times x_{\left(i+\Delta x_{\left(i,j\right)},j+\Delta y_{\left(i,j\right)}\right)} \end{array}$$

Similarly, i, j traverse the set (-1,0,1).

### 3.2 Information selection feature fusion

**Figure 2:**
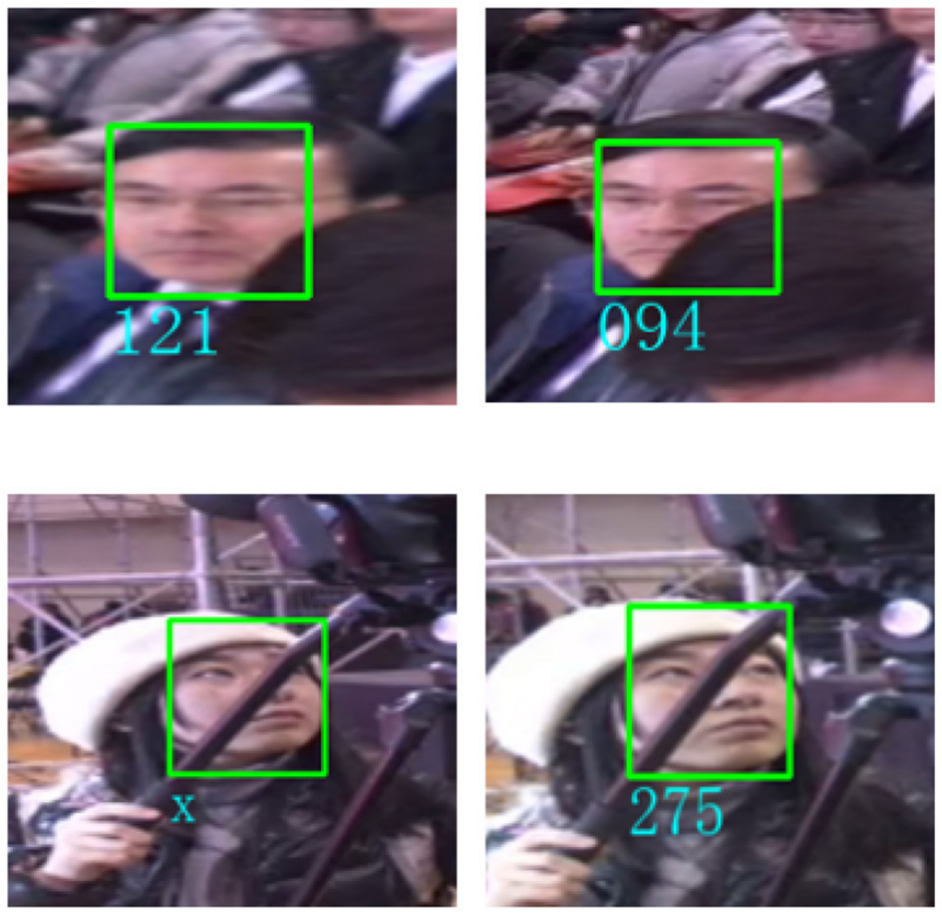
Identity mismatch in left and right eye image recognition of the same person.

In panoramic stereoscopic videos, there may be differences between the two videos captured from different angles, where faces are presented in various postures and may also be partially occluded. In this case, if the faces are detected in the upper and lower channels of panoramic stereoscopic video separately, there may be situations where the same person's facial images in the two channels are recognized as different identities due to the above differences. Figure 2 shows the problem that occurred during the recognition process. Therefore, for panoramic stereoscopic videos, it is necessary to fully utilize the common information of the faces in the upper and lower videos, and use the facial images in both channels as input to the model. After preliminary feature extraction, fusion is performed, and then deep feature extraction is performed to ultimately obtain the facial feature vector.

**Figure 3:**
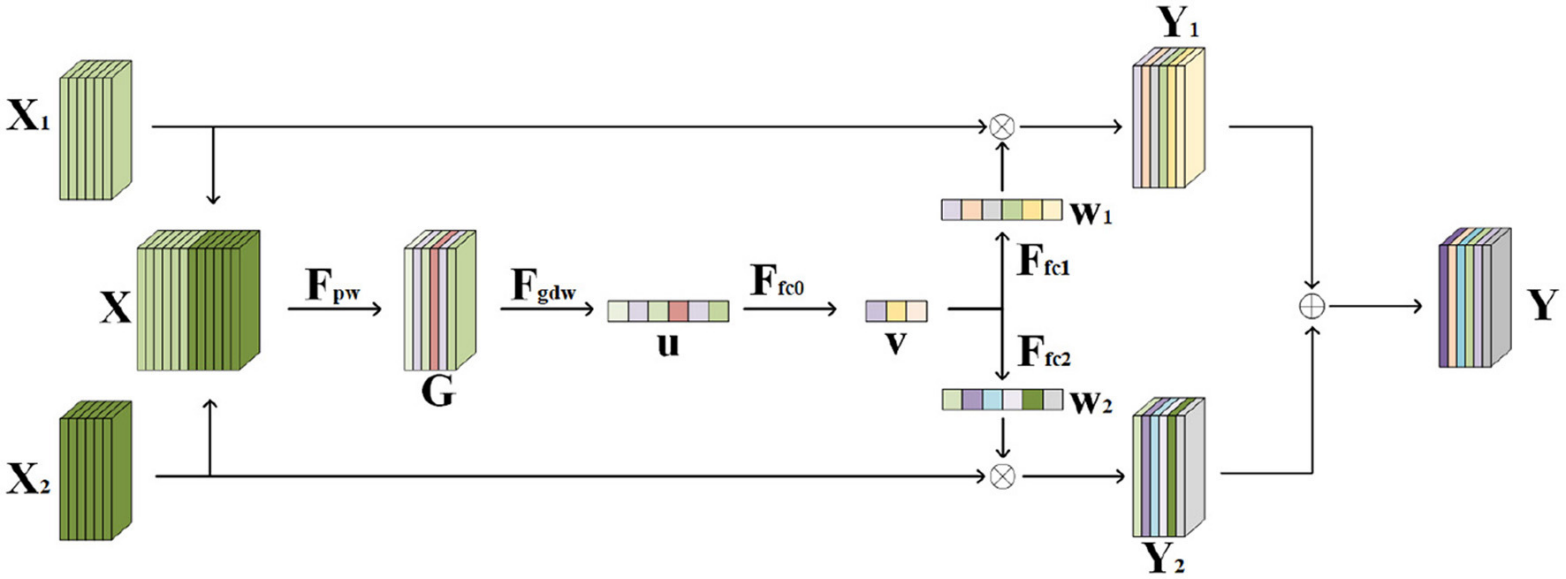
Information selection feature fusion structure.

The inputs to the network are two face images of left and right eyes, so feature fusion of the two inputs needs to be performed in the network. There are many commonly used feature fusion methods for the same size feature maps, such as the ResNet network which directly sums the feature maps numerically. However, when the input is a left and right eye binocular face image, the two input images have partially the same feature information and also each has information that is not present in the other input, so these methods lose most of the spatial information or produce information redundancy, so the feature map information needs to be selected at the time of feature fusion. As Figure 3 demonstrates the flow of the information selection feature fusion (SF) structure, is mainly divided into three steps: splicing, selection, and fusion.

Assume that the feature maps of the two inputs after shallow feature extraction are *X*_1_ and *X*_2_, and the height, width, and number of channels of both are *H, W*, and *C*. The two feature maps are first spliced dimensionally (Equation 5):


(5) $$\begin{array}{l} X=X_{1}+X_{2} \end{array}$$

After splicing, the height and width of the feature map remain unchanged, and the number of channels becomes twice as many as the original *2C*. At this time, the feature map *X* contains the common information, as well as the unique information of the left and right eye face images, and the duplicated common information, will become redundant information, so it is necessary to preliminarily screen the redundant information through the 1 × 1 point convolution (Equation 6):


(6) $$\begin{array}{l} G_{n\left(i,j\right)}=F_{pw}\left(X_{\left(i,j\right)}\right)=\overset{2C}{\underset{k=1}{\sum }}W_{n,k}\times X_{k\left(i,j\right)} \end{array}$$

(7) $$\begin{array}{l} G=\left\{G_{1},G_{2},G_{3},\cdots \;,G_{n}\right\} \end{array}$$

where *G*_*n*(*i, j*)_ is the element with coordinate (*i, j*) in the nth channel of the output feature map *G* (Equation 7), *W*_*n, k*_ is the weight in the convolution kernel of the nth output feature map channel corresponding to the kth input feature map channel, and *X*_*k*(*i, j*)_ is the element with coordinate (*i, j*) in the kth input channel. The output feature map width and height are unchanged, and the number of channels *n* is changed back to the number of input feature map channels *C* before splicing.

In the selection step, the feature map first needs to be downsampled by shrinking the height and width to 1 into a feature vector, using a single value to represent the spatial information within the entire channel. In convolutional neural networks, the feature map is usually downsampled using a pooling operation, which is computationally simple and introduces no additional parameters but can have some problems. When maximum pooling is used, the maximum value of the entire channel's features is used to represent that channel's information, which discards the rest of the features and loses a lot of spatial information. When average pooling is used, the average of the features in the channel is used as a representative, but the importance of the features is different from each other, and average pooling does not fully take into account the contribution weight of each feature, which will reduce the proportion of important information. Therefore, the feature map can be downsampled using global deep convolution, which allows the network to autonomously assign the importance weight of each feature without causing too much information to be lost. The process of global deep convolution is (Equations 8, 9):


(8) $$\begin{array}{l} u_{c}=F_{gdw}\left(G_{c}\right)=\overset{W}{\underset{i=1}{\sum }}\overset{H}{\underset{j=1}{\sum }}W_{c\left(i,j\right)}\times G_{c\left(i,j\right)} \end{array}$$

(9) $$\begin{array}{l} \vec{u}=\left\{u_{1},u_{2},u_{3},\cdots \;,u_{c}\right\} \end{array}$$

where *u*_*c*_ is the value of the cth channel after global deep convolution, which represents the information of the cth channel. *W*_*c*_ is the deep convolution convolution kernel parameter matrix of the cth channel.

Global deep convolution compresses the spatial information within each channel but does not fuse interactions between channels. Therefore, further compression of the feature vectors is needed through a fully connected layer utilizing the feature information between channels (Equation 10):


(10) $$\begin{array}{l} \vec{v}=F_{fc0}\left(\vec{u}\right)=\mathrm{ReLU}\left(W_{0}\times \vec{u}\right) \end{array}$$

where *W*_0_ is the parameter matrix of the fully connected layer and the dimension of the output feature vector $\vec{v}$ is half the dimension of the input $\vec{u}$, *c*/2.

After that, the compressed feature vectors need to be mapped to feature vectors with the same dimensions as before compression as weights for different channels in each input:


(11) $$\begin{array}{l} \vec{w_{1}}=F_{fc1}\left(\vec{v}\right)=\mathrm{Sigmoid}\left(W_{1}\times \vec{v}\right) \end{array}$$

(12) $$\begin{array}{l} \vec{w_{2}}=F_{fc2}\left(\vec{v}\right)=\mathrm{Sigmoid}\left(W_{2}\times \vec{v}\right) \end{array}$$

where *W*_1_ and *W*_2_ are the parameter matrices of the two fully connected layers, and $\vec{w_{1}}$ (Equation 11) and $\vec{w_{2}}$ (Equation 12) are the weight vectors of dimension *c*. Since the activation function is a Sigmoid activation function, the weight size is between 0 and 1.

After obtaining the weight vectors, it is necessary to let the input two-channel feature maps *X*_1_ and *X*_2_ select information according to the weight vectors $\vec{w_{1}}$ and $\vec{w_{2}}$. At this time, the channel where the redundant information is located corresponds to lower weights, and the exclusive information has higher weights, so that the selection of information and the finalization of feature fusion can be achieved:


(13) $$\begin{array}{l} Y_{1c}=X_{1c}\times w_{1c} \end{array}$$

(14) $$\begin{array}{l} Y_{2c}=X_{2c}\times w_{2c} \end{array}$$

(15) $$\begin{array}{l} Y_{c}=X_{1c}+X_{2c} \end{array}$$

Where *X*_1*c*_ (Equation 13) and *X*_2*c*_ (Equation 14) are the feature maps of the input feature maps *X*_1_ and *X*_2_ in the cth channel, respectively, *Y*_*c*_ (Equation 15), *Y*_1*c*_, *Y*_2*c*_ are the same, and *w*_1*c*_ and *w*_2*c*_ are the weights of the corresponding channels, respectively.

Finally, after the two feature maps are added together, the information selection feature fusion is completed. The fused feature map *Y* realizes the information selection of the input two feature maps, filtering out the shared redundant information and retaining the unique features.

### 3.3 Interchannel message redistribution

Deeper in the network, the feature map is obtained by splicing two face images through the channel dimensions, and thus more attention needs to be paid to the inter-channel information. The Squeeze and Excitation (SE) structure can redistribute the information weights between the channels, allowing the network to autonomously learn the importance of different channels from the perspective of the whole channel and modify the weights of each channel.

The idea behind the implementation of the SE structure is to first globally pool the feature map by reducing both the width and height to 1, leaving the number of channels unchanged, and using a single value to represent the information of the entire channel, the feature map becomes a feature vector. Next, the feature vector is input to a fully connected layer, and the output of the fully connected layer becomes *1/m* of the original number of channels, this step is called compression. Subsequently, the compressed feature vector is scaled through a fully connected layer and the number of output channels is increased to *m* times, i.e., the same as the number of input channels of the SE structure, and then it is nonlinearly transformed by a Sigmoid function, and hence this step is called excitation. Since the input feature maps are scaled to feature vectors of the same dimension, the effect of using a 1 × 1 point convolution in compression and excitation is the same as using a fully connected layer. Finally, the reassigned weight values corresponding to each channel are obtained after the excitation and the feature maps after multiplying each channel in the input feature maps of the SE structure by the weight values of the corresponding channels are used as the outputs of the SE structure to be sent to the subsequent networks.

To better compress the information in each channel and make the compressed data more representative, global pooling is replaced with global deep convolution. Every information within the channel in the global deep convolution is involved in the operation, which can make the output more representative of the features of the whole channel. Although an additional number of parameters is introduced, the number of parameters cannot be compared with the number of parameters in the fully connected layer in compression and excitation. At the same time, global deep convolution can bring better results.

The SE structure mainly redistributes information between channels, so it is better to set it after feature extraction. Combined with the inverted residual structure, the SE structure is set after the 3 × 3 deep convolution feature extraction and before the 1 × 1 convolution dimensionality reduction.

### 3.4 DCM2Net model

To fully utilize the face information from different angles, the DCM2Net network takes in two face images for the left and right eyes and performs simultaneous feature extraction. However, this can result in a high number of parameters. To mitigate this issue, the first few layers of the network, which perform shallow feature extraction, can share the same inverted residual module parameters as the shallow features in the left and right eye face images are similar and overlapping.

**Figure 4:**
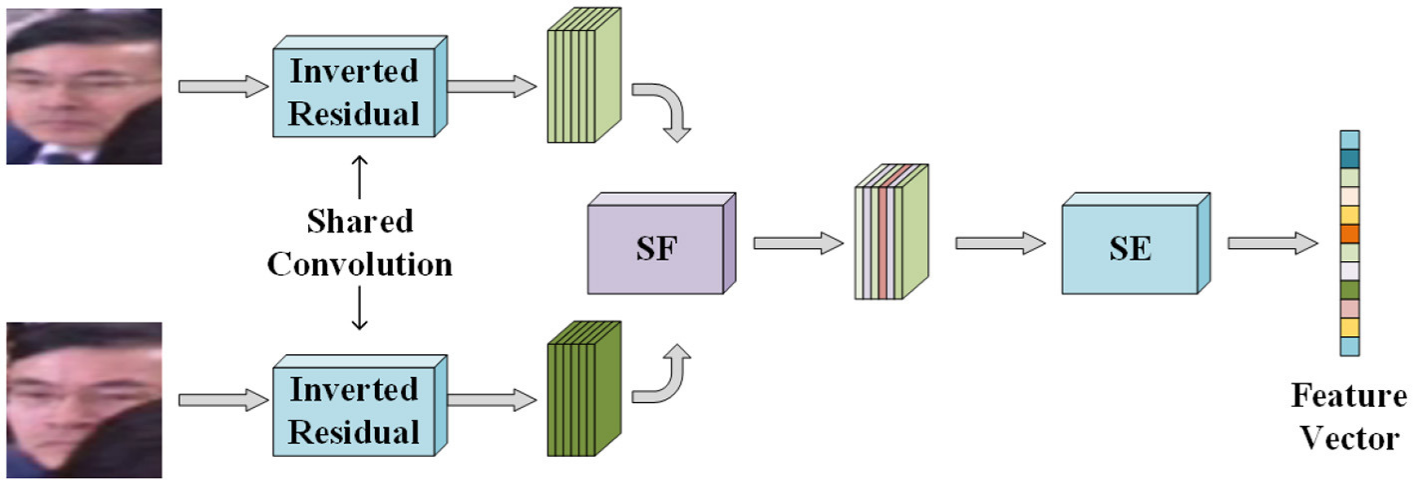
DCM2Net network model structure. The input of the network is left and right binocular face images, and the output of the network is 128 dimensional feature vector.

The shallow feature extraction of the two inputs is done through shared inverted residual modules. The two feature maps are then stitched together in the channel dimension, which results in a feature map that contains the full information of both left and right binocular face images. This stitched feature map includes both common and unique information, and 1x1 point convolution is used to integrate the information between channels, retaining more useful features and reducing the amount of data and number of parameters. The DCM2Net network and its specific parameters are shown in Figure 4, Table 1 respectively.

**Table 1:** DCM2Net network parameters.

**Operation name**	**Input size**	**Pace**	**Left-hand flow**	**Right-hand flow**
Shared Conv1	112^2^×3	2	$\left[\begin{array}{c} 3\times 3dw,3\\ 1\times 1,32 \end{array}\right]\times 1$	$\left[\begin{array}{c} 3\times 3dw,3\\ 1\times 1,32 \end{array}\right]\times 1$
Shared Btk1	56^2^×32	2	$\left[\begin{array}{c} 1\times 1,64\\ 3\times 3dw,64\\ 1\times 1,32 \end{array}\right]\times 2$	$\left[\begin{array}{c} 1\times 1,64\\ 3\times 3dw,64\\ 1\times 1,32 \end{array}\right]\times 1$
Shared Btk2	28^2^×64	2	$\left[\begin{array}{c} 1\times 1,128\\ 3\times 3dw,128\\ 1\times 1,64 \end{array}\right]\times 1$	$\left[\begin{array}{c} 1\times 1,128\\ 3\times 3dw,128\\ 1\times 1,64 \end{array}\right]\times 1$
SF	(14^2^×64) × 2	1	SF, 64	
Btk1	14^2^×128	1	$\left[\begin{array}{c} 1\times 1,128\\ 3\times 3dw,128\\ 1\times 1,128 \end{array}\right]\times 4$	
Btk2	14^2^×128	2	$\left[\begin{array}{c} 1\times 1,512\\ 3\times 3dw,512\\ 1\times 1,256 \end{array}\right]\times 1$	
DcBtk(SE)	7^2^×256	1	$\left[\begin{array}{c} 1\times 1,512\\ 3\times 3dw,512\\ 1\times 1,1024\left(\text{offset}\right)\\ 3\times 3dw,512\\ SE\\ 1\times 1,256 \end{array}\right]\times 2$	
Conv1	7^2^×256	1	1 × 1, 512	
Conv2	7^2^×512	1	7 × 7*dw*, 512	
Conv3	1^2^×512	1	1 × 1, 128	

## 4 Panoramic stereoscopic video live streaming and face recognition system

**Figure 5:**
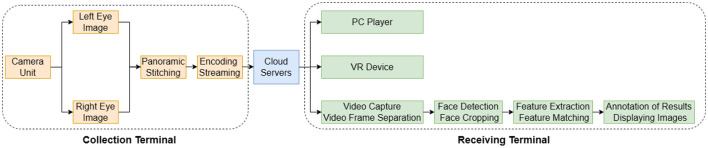
Panoramic stereoscopic video live system process.

Figure 5 is the functional module diagram of the panoramic stereoscopic video live broadcast system. The system describes all the functional modules of the panoramic stereoscopic video live broadcast and face recognition system, showing the system architecture from image acquisition, to panoramic video splicing and encoding transmission, to the final user to receive and watch, and face recognition. The system is mainly divided into three major functional modules: the acquisition side, the cloud forwarding side, and the receiving side.

### 4.1 System acquisition side

**Figure 6:**
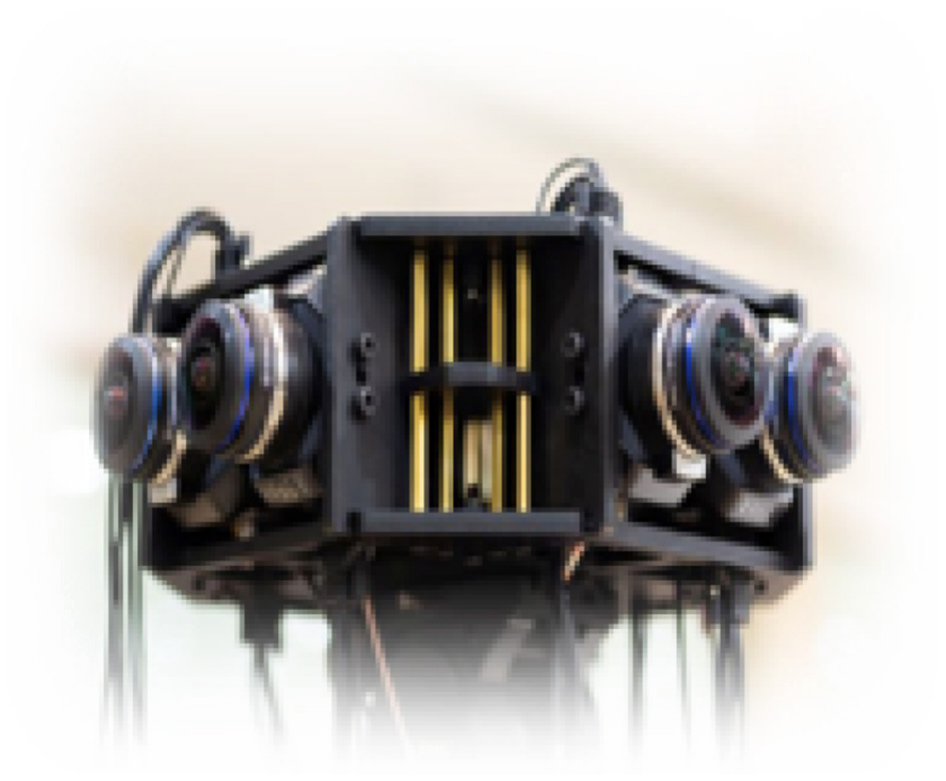
Acquisition end camera sets.

The acquisition end of this system consists of 8 identical fisheye cameras, which are divided into 4 groups, each group contains 2 fisheye cameras, one left and one right constitutes a binocular camera, and they have the same shooting direction. The shooting directions of the two neighboring groups of binocular cameras form a right angle, so the 4 groups of cameras can cover a 360-degree range to achieve the effect of panorama, and the system is built as shown in Figure 6. The model of the fisheye camera is iZugar MKX22 on Blackmagic micro Studio 4K, and the images captured by the camera will be transferred to the server through the SDI interface with the capture card for subsequent operations.

The system uses the PT Gui tool to stitch together panoramic image templates. A single frame captured simultaneously by the eight-eye lens is first acquired, and then the neighboring camera images are selected to match the feature points. Since a set of binocular lenses is used for acquisition in each direction, the left and right eye images need to be spliced separately. Eventually, the four left-eye images are stitched together to become the left-eye panoramic picture and the four right-eye images are stitched together to become the right-eye panoramic picture. The two panoramic images are connected as a panoramic image stitching template, according to which the binocular panoramic video can be stitched in real time.

The system uses the VR Studio tool to read the real-time images from each camera, remap the panoramic image stitching template generated by PT Gui, and get the real-time panoramic stereoscopic video. In the configuration page, customize the settings of video resolution, bit rate, video frame rate, and other live parameters, and fill in the live address to achieve the encoding push stream.

### 4.2 Cloud forwarding module

Currently in a variety of network video sites in most of the H.264 standard video encoding transmission, but the panoramic stereoscopic video clarity up to 8K, the use of H.264 encoding still needs to transmit an extremely large amount of data, so the system adopts H.265 encoding format. The realization of panoramic stereoscopic video live transmission, the need to capture the end of the video stream pushed to the cloud server after forwarding, users outside the LAN can receive to watch, the system selected RTMP protocol for transmission. Various streaming media servers support RTMP protocol, and this system uses the SRS (Simple Rtmp Server) server as the streaming media server.

### 4.3 Video reception and display

After the video stream is pushed to the SRS server, it can be accessed anywhere on the Internet to pull the stream for decoding and viewing, and using different forms of playback will also provide a different viewing experience. Usually, people watch videos by using players. Take the third-party player PotPlayer as an example, after combining the H.265 decoder, you can watch it by entering the live streaming address configured by the SRS server in the player. Another way of viewing is to use a VR device, which is the most suitable playback device for viewing panoramic videos, such as the Pico all-in-one.

### 4.4 Face recognition system

After acquiring the panoramic stereoscopic video frame, face detection needs to be performed on the current frame. If the image contains a human face, the area where the face image is located is cropped, the cropped face image is scaled to a resolution of 112 × 112, and the upper and lower face images are paired to record two face images of the same person for use in face recognition.

The system uses DCM2Net to extract features when the input is the paired upper and lower face images of the same person with a resolution of 112 × 112 and the output is a 128-dimensional feature vector. Subsequently, the feature vectors corresponding to each face need to be matched in the face database. The matching method is to calculate the Euclidean distance between the feature vectors of the face to be recognized and the feature vectors in the database. After the recognition is finished, the matched identity ID is displayed below the corresponding face position. To ensure the real-time nature of live video face recognition, the video can be sampled frames, and the recognition can be carried out once every interval of a fixed number of frames, which does not affect the smoothness of the live video playback, but also avoids the repetitive recognition of the faces that do not change much in the video frames that are close to each other.

The face database needs to recognize the identities to be recognized that may appear in the video in advance, and each person can enter multiple face features, including front face, side face, different expressions, etc., to simulate various scenarios that may appear in the video. The recording process is similar to the face recognition process, firstly, the face image is cropped by face detection alignment, secondly, the face features are extracted, the feature vectors output from the network are stored in the database, and the corresponding identities are recorded. The system supports adding, modifying, and deleting operations of faces and identities in the face database at any time.

## 5 Results

### 5.1 Dataset

**Figure 7:**
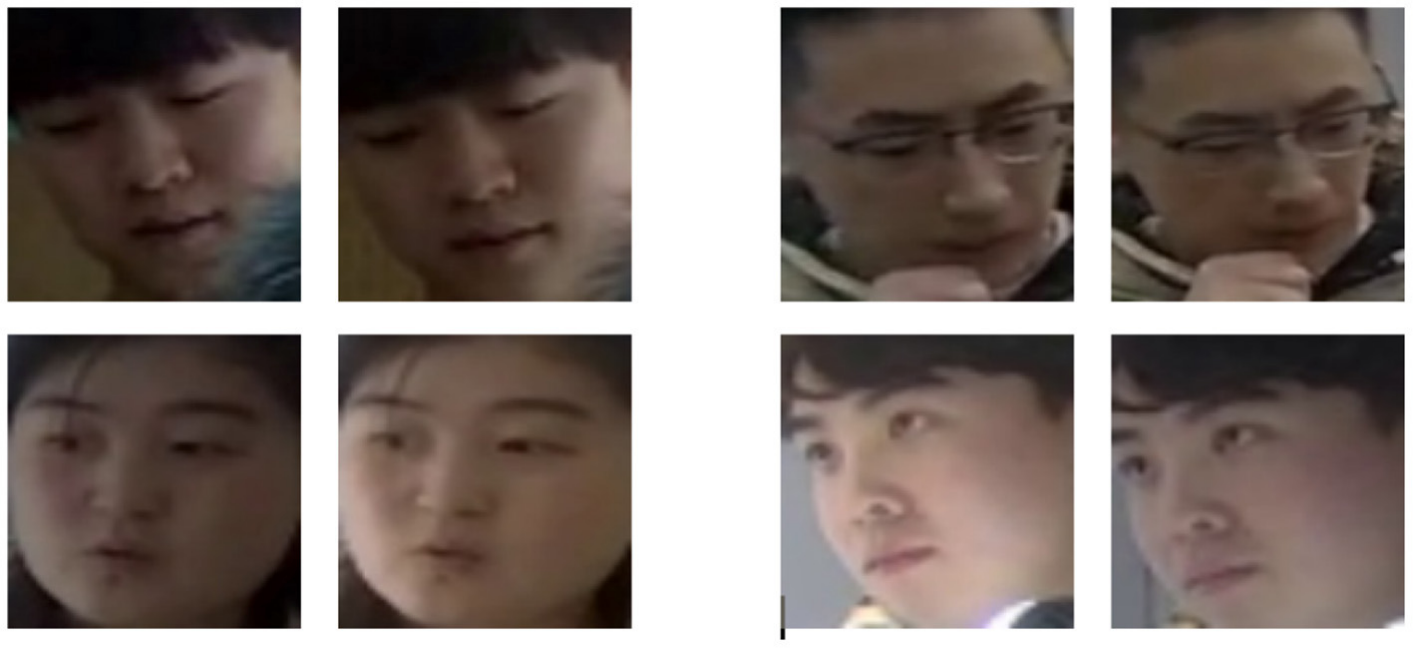
Some images from the PSD dataset.

In this paper, a new Panoramic Binocular Face Dataset (Panoramic Stereo Dataset, PSD) is constructed. This dataset is partly collected from the web containing binocular images and videos of faces and partly collected by the Panoramic Stereo Video Acquisition System. After the same face detection and cropping, the left and right eye face images of the same person are paired to be used for training and testing. The dataset contains a total of 5236 face images of 40 people, with every two constituting a pair of binocular face images. 80% of the data in the dataset is randomly selected for training and the remaining 20% is used for testing, Figure 7 illustrates some of the PSD dataset images. The test dataset consists of LFW (Huang et al., 2008), CFP (Sengupta et al., 2016), AgeDB (Moschoglou et al., 2017), VGGFace2 (Cao et al., 2018), and CPLFW (Knoche et al., 2021).

### 5.2 Model training process

**Figure 8:**
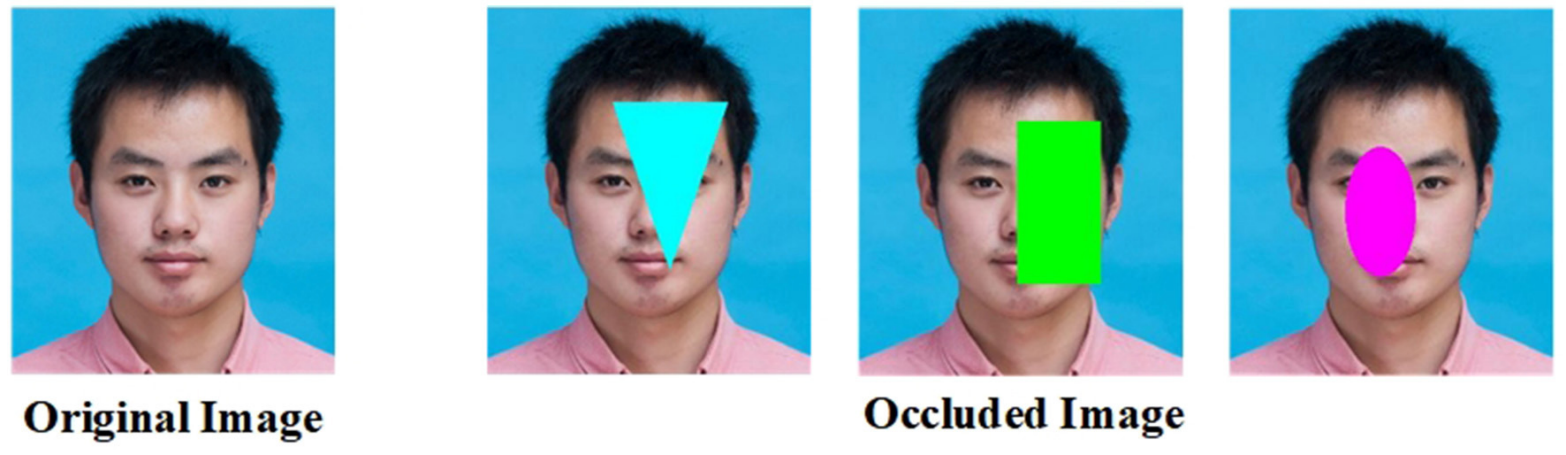
Monocular face dataset to generate occlusion images.

The system first uses the MS1M face dataset to pre-train the DCM2Net network, followed by fine-tuning using the PSD dataset. Since MS1M is a monocular face dataset, during pre-training, one of the inputs of the network directly uses the face images in the dataset, and the first input face image is added with random occlusion as the second input to achieve the effect of simulating the different inputs, as shown in Figure 8. After generating the simulated occlusion samples, data enhancement processes, including image flipping, rotation, cropping, and random blurring are applied to both input data to enhance the generalization of the network model.

**Figure 9:**
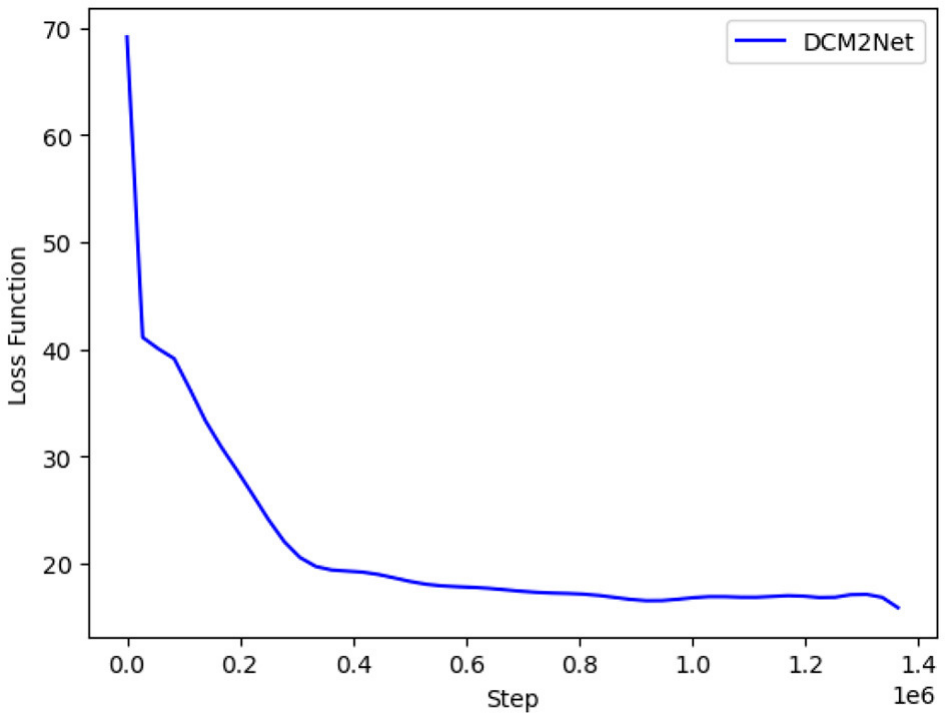
Variation of the DCM2Net loss function.

During training, the training epoch was set to 15, the batch input size was 64, the initial learning rate was 0.1, and the learning rate was reduced to one-tenth of the previous rate when the period epoch reached 2, 5, 8, and 11, respectively, and the model was accelerated to convergence using the Adam optimizer. The changes in the loss function were recorded during training, as shown in Figure 9.

### 5.3 DCM2Net results

To evaluate the effectiveness of the DCM2Net model, tests were performed on multiple datasets. When the model was tested on the binocular face dataset, two pairs of binocular face images were directly input to compute the feature vectors; when it was tested on the monocular face dataset, the original face image and the current partially occluded image of the face were input separately. The binocular face dataset contains only the PSD dataset and the others are monocular face datasets. The experimental results are shown in Table 2.

**Table 2:** Comparison of DCM2Net experiments.

**Models**	**LFW(%)**	**CFP(%)**	**AgeDB30(%)**	**PIFI(%)**	**PSD(%)**
ArcFacem (Deng et al., 2019)	99.53	95.56	95.15	93.46	89.26
MixFaceNet (Boutros et al., 2021)	99.68	-	97.05	93.18	88.62
ShuffleFaceNet (Martindez-Diaz et al., 2019)	99.45	96.04	96.33	92.64	88.57
MobileFaceNets (Chen et al., 2018)	99.47	91.86	96.00	93.23	88.71
DCMNet-22dc (Zhang et al., 2021)	99.52	92.03	95.67	94.92	89.19
DCMNet-4dc (Zhang et al., 2021)	99.51	93.16	96.27	94.37	89.74
DCM2Net	99.46	94.22	91.24	93.85	92.45

From the experimental results, it can be seen that the result using DCM2Net in the monocular face image dataset LFW is 99.16%, which is slightly lower than the rest of the methods, but still possesses a high accuracy. The result on CFP is 92.22%, which is behind ArcFace's 95.56% and ShuffleFaceNet's 96.04%. In AgeDB30, a dataset with a large age gap, the result of DCM2Net is 91.24%, which still has much room for improvement. In the panoramic face hybrid dataset PIFI, DCM2Net possesses 93.85% accuracy, which is 1.07 percentage points lower than the DCMNet model, but both are higher than the remaining methods. This is because both models, DCMNet and DCM2Net, introduce deformable convolution, and thus can fully extract deformed face features. Overall, DCM2Net is more general for monocular face images, so the network is not suitable for face recognition in monocular videos. In the binocular face dataset PSD, PSFNet has the highest accuracy of 92.45%. Combining the results, it can be concluded that DCM2Net can fully utilize the information from the left and right binocular face images of the same person, and is, therefore, suitable for face recognition tasks in panoramic stereoscopic videos.

To investigate whether the SE structure is effective in fusing inter-channel information and extracting features from different angles of the face, DCM2Net without the SE structure is also trained for comparisons. The model also uses binocular face images as input, and after feature fusion using 1 × 1 convolution, only the information is extracted through the deformable inverse residual structure without SE, and the final feature vector is output. A test was also performed using the DCMNet-4dc model as a reference. For testing, a set of test images contains two pairs of left and right-eye face images, and for the DCM2Net network, only the Euclidean distance between the feature vectors of these two pairs of face images is computed. For the DCMNet-4dc model, each of the four face images will be computed with the remaining images. The experimental results are shown in Table 3.

**Table 3:** Comparison in the PSD dataset.

**Models**	**PSD(%)**	**Model size(M)**	**Number of entries(M)**
DCMNet-4dc	89.74	7.2	1.8
DCM2Net	92.45	6.9	1.7
DCM2Net(No SE)	90.79	6.3	1.5

From the experimental results, it can be seen that the DCM2Net with two inputs has higher accuracy in the binocular face dataset PSD compared to the DCMNet with single input, while the DCM2Net with SE structure has better recognition results due to its more focus on inter-channel information. Moreover, the sharing of parameters between the two inputs of the shallow network allows for a smaller number of parameters and a smaller model size.

### 5.4 Face recognition experiment results

To evaluate the accuracy of DCM2Net for face recognition in panoramic stereoscopic videos, the system captured and recorded videos in four different scenarios, including the laboratory, live broadcasting room, conference room, and campus. At the same time, a segment of about 15 seconds in length was intercepted for each video, and conditions such as clear video images and large facial changes were satisfied during the interception. In the same video, the content of the screen between several consecutive frames changes very little, so experiments were performed to recognize the video clips every second.

Face detection is the first step of a face recognition system, a good face detection method can detect more accurate and high-quality faces, to provide a guarantee for subsequent face recognition. The experiment selects CenterFace (Xu et al., 2020) and RetinaFace (Deng et al., 2020) two face detection methods for testing respectively. The experimental results are shown in Table 4, where the total number of occurrences is the total number of occurrences in all 15 to-be-recognized frames in the video clip, with repeated counts of the same person appearing multiple times. The number of correct detections is the number of correctly detected face images and the number of detection errors is the number of non-face targets detected as faces. CF denotes the detection of the CenterFace method, and RF denotes the detection of the RetinaFace method. Overall, the CenterFace method is slightly better than the RetinaFace method, so CenterFace is chosen as the face detection part of the system.

**Table 4:** Combined detection situations for CF and RF.

	**Laboratory**		**Broadcasting room**		**Conference room**		**Campus**	
	**CF**	**RF**	**CF**	**RF**	**CF**	**RF**	**CF**	**RF**
Times	30	30	120	120	150	150	326	326
Correct	28	28	62	58	94	88	114	104
Incorrect	0	0	1	0	0	1	3	4

After CenterFace face detection, DCM2Net is used to recognize the detected faces in each scene. The selection of the judgment threshold affects the result of face recognition, if the threshold is large, there will be many times when the recognition of the identity is wrong; if the threshold is small, there will be many times when the information of the person to be recognized is already in the database, but the recognition result is not in the database. According to the results in Table 2, the threshold value of 0.9 when the model has the highest test accuracy in the PSD dataset is finally selected as the judgment threshold for this experiment, and the experimental results are shown in Table 5.

**Table 5:** DCM2Net recognition situation.

	**Laboratory**	**Broadcasting room**	**Conference room**	**Campus**
Total occurrences	30	120	150	326
Correct detections	28	58	88	104
Correct recognitions	14	26	38	42
Incorrect recognitions	0	2	5	7
Unmatched detections	0	1	1	3
Recognition accuracy	100%	89.7%	86.4%	80.8%

**Figure 10:**
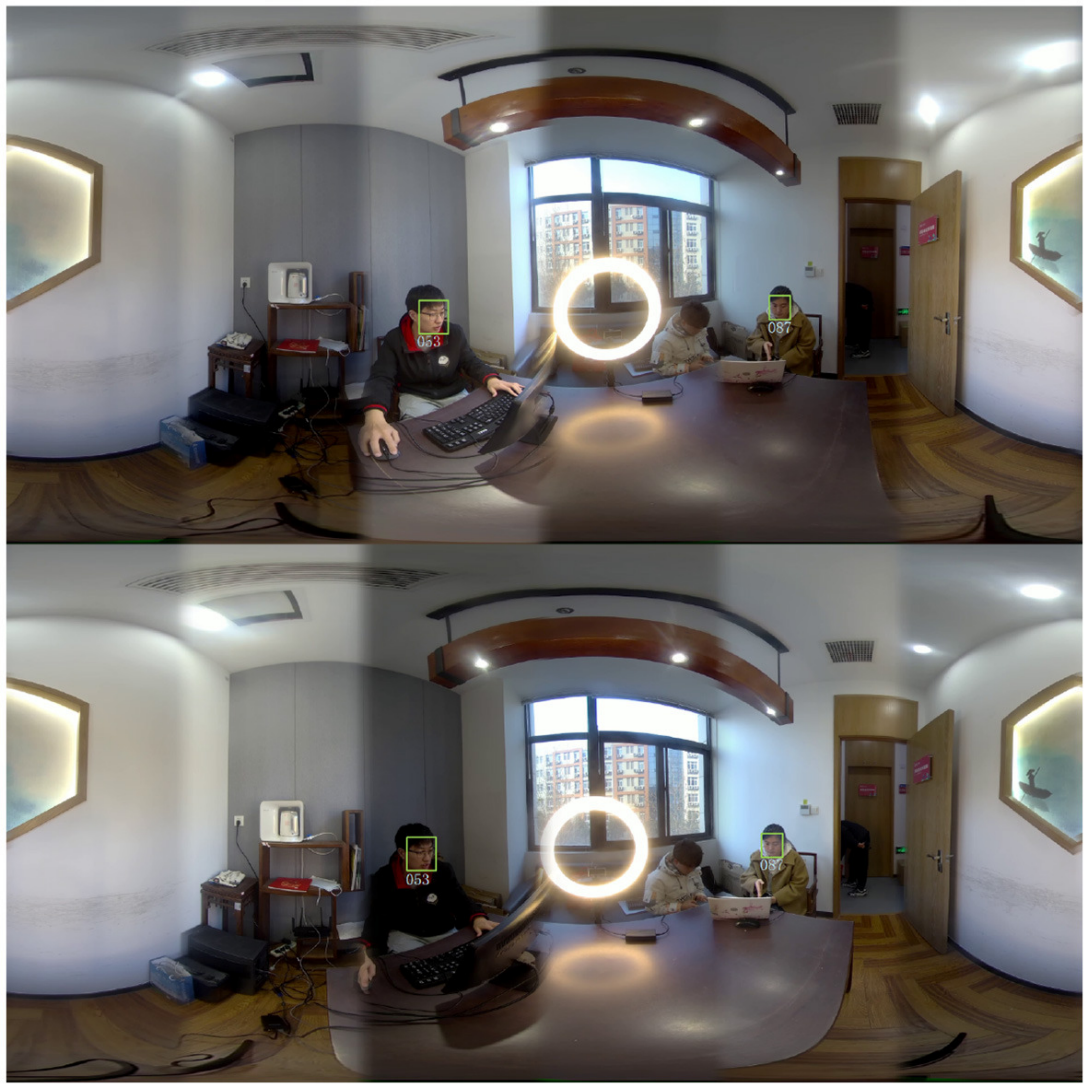
Face recognition results of live streaming room.

Among them, the number of correct recognitions is the number of correct identity matches in the database, the number of recognition errors is the number of incorrect identity matches in the database, and the number of unmatched to is the number of Euclidean distances between the current face feature vectors and all the existing feature vectors in the database are greater than the judgment threshold. Since detection is performed on all faces in the panoramic stereoscopic video, and recognition uses two face images from the left and right eyes to match an identity, the sum of the number of correctly recognized, the number of incorrectly recognized, and the number of unmatched-to is one-half of the number of correctly detected. For example, Figure 10 shows the result of face recognition in the live streaming room.

There are large differences in the recognition accuracy in different scenes, mainly caused by multiple factors such as face image quality, face pose, and face occlusion. In the laboratory shooting scene, there is only one person, there is not much change in the face, so all of them are recognized correctly. In the rest of the indoor scenes, the quality of the face image is better, and there are some changes in the face posture, so the recognition effect is better. However, in the outdoor scenes, the face images are of low resolution, and low quality, and most of them are side faces with partial occlusion, so the recognition effect is more general.

## 6 Conclusion

To address the issues of barrel distortion and binocular camera shooting angle, we propose a solution called DCM2Net to improve the accuracy of facial recognition in panoramic stereoscopic videos. We also designed a panoramic stereoscopic video broadcasting system using four sets of binocular cameras for image acquisition, panoramic stitching, coding, and streaming. We also created a panoramic data set PSD based on a panoramic stereo video system. The results indicate that our model performs well not only on traditional datasets but also on panoramic datasets.

## Data availability statement

The raw data supporting the conclusions of this article will be made available by the authors, without undue reservation.

## Ethics statement

Written informed consent was obtained from the individual(s) for the publication of any potentially identifiable images or data included in this article.

## Author contributions

DZ: Conceptualization, Methodology, Project administration, Visualization, Writing – original draft, Writing – review & editing. WT: Data curation, Formal analysis, Funding acquisition, Investigation, Writing – review & editing. YW: Resources, Software, Supervision, Validation, Writing – original draft. CT: Data curation, Investigation, Visualization, Writing – review & editing.
